# Tau pathology in epilepsy: emerging mechanisms and translational opportunities

**DOI:** 10.1093/brain/awag108

**Published:** 2026-03-23

**Authors:** Arjune Sen, Xin You Tai, Aristea Galanopoulou, Maria Thom, Eleonora Aronica, Lucy Vivash, Martin Hardmeier, Action Amos, Stephan Rueegg, Matthias Koepp, Yaroslav Winter, Christoph Helmstaedter, Jeffrey L Noebels, Hilal A Lashuel, Terence J O’Brien

**Affiliations:** Oxford Epilepsy Research Group, Nuffield Department of Clinical Neuroscience, University of Oxford, Oxford OX3 9DU, UK; Division of Clinical Neurology, John Radcliffe Hospital, Oxford University Hospitals NHS Foundation Trust, Oxford OX3 9DU, UK; Centre for Global Epilepsy, Wolfson College, Oxford OX2 6UD, UK; Oxford Epilepsy Research Group, Nuffield Department of Clinical Neuroscience, University of Oxford, Oxford OX3 9DU, UK; Division of Clinical Neurology, John Radcliffe Hospital, Oxford University Hospitals NHS Foundation Trust, Oxford OX3 9DU, UK; Saul R, Korey Department of Neurology, Laboratory of Developmental Epilepsy, Isabelle Rapin Division of Child Neurology, Dominick P. Purpura Department of Neuroscience, Albert Einstein College of Medicine, Bronx, NY 10461, USA; Department of Epilepsy, UCL Queen Square Institute of Neurology, London WC1N 3BG, UK; Department of (Neuro)Pathology, Amsterdam UMC, University of Amsterdam, Amsterdam Neuroscience, Amsterdam 1105 AZ, The Netherlands; The Department of Neuroscience, School of Translational Medicine, Monash University, Melbourne, Victoria 3004, Australia; Department of Neurology, Alfred Health, Melbourne, Victoria 3004, Australia; Department of Neurology, Epilepsy and Sleep Unit, Medical Faculty of the University of Basel, University Hospital Basel, Basel CH-4031, Switzerland; Centre for Clinical Brain Sciences, University of Edinburgh, Edinburgh EH16 4SB, UK; Department of Neurology, Epilepsy and Sleep Unit, Medical Faculty of the University of Basel, University Hospital Basel, Basel CH-4031, Switzerland; Department of Epilepsy, UCL Queen Square Institute of Neurology, London WC1N 3BG, UK; Department of Neurology, Saarland University Medical Center, University of Saarland, Homburg 66421, Germany; Department of Epileptology, University Hospital Bonn, Bonn 53127, Germany; National Center for Epilepsy, Oslo University Hospital, Oslo 0424, Norway; Developmental Neurogenetics Laboratory, Department of Neurology, Baylor College of Medicine, Houston, TX 77030, USA; Laboratory of Molecular and Chemical Biology of Neurodegeneration, Institute of Bioengineering, School of Life Sciences, Ecole Polytechnique Fédérale de Lausanne, Lausanne 1015, Switzerland; Weill Cornell Medicine Qatar, Education City, Qatar Foundation, Doha, Qatar; Department of Neurology, Weill Cornell Medicine, New York, NY 10065, USA; The Department of Neuroscience, School of Translational Medicine, Monash University, Melbourne, Victoria 3004, Australia; Department of Neurology, Alfred Health, Melbourne, Victoria 3004, Australia

**Keywords:** cognition, dementia, global challenge, neurodegeneration, seizures

## Abstract

The onset of epilepsy in adulthood occurs most commonly after 55 years of age. Given the ageing global population, this disorder represents an increasing burden on healthcare and society. The bidirectional link between epilepsy and dementia is a focus of intense research with underlying tau pathology highlighted as a potential mechanistic link.

In this review, we examine the evidence for tau-related neurodegenerative processes in epilepsy beginning with how changes in biochemical and structural properties of the tau protein can lead to abnormal phosphorylation and pathological aggregation. We consider the role of tau in seizure occurrence and cognitive difficulties in experimental animal epilepsy models to human epileptic syndromes. Seizure prevalence is evaluated across established primary and secondary tauopathies to understand the associated hyperexcitability phenotype. We discuss the use of neurophysiology, metabolic imaging and novel fluid biomarkers as non-invasive measures of potential underlying neurodegeneration in epilepsy. It may, for example, be that these can be combined with remote measures of cognition and other physiological parameters to provide accurate longitudinal monitoring of cognition and underlying pathology. We also explore clinical trials that have targeted pathological tau accumulation in neurodegenerative conditions and consider an ongoing clinical study with sodium selenate, an enhancer of protein phosphatase enzyme PP2A, in people with epilepsy. These efforts signify a novel disease-modifying era with treatments that reduce seizures and modify cognitive outcomes in people with epilepsy.

Our analysis of the literature underscores the need for more in-depth characterization of tau pathology, at biochemical and structural levels in brain tissue and peripheral samples from people with epilepsy, as an important step to deciphering the role of tau in the pathogenesis of epilepsy and related disorders. Examining the relationships between tau pathology and cognitive impairment in those with epilepsy provides critical perspectives on potential causal tau pathomechanisms that may have important roles in epileptogenesis and dementia.

## Teaser

Epilepsy is most common in older adults and has a bidirectional relationship with dementia. Sen *et al.* explore tau pathology as a potential underlying mechanism, drawing on biochemical findings, animal models and human epileptic syndromes, highlighting tools and treatments that could modify disease progression and improve outcomes.

## Introduction

The global population is ageing,^1^ particularly in low resource settings, with the ageing population rate in Africa being three times greater than in Europe or the USA.^2^ Similar changes are evident across Asia. In Malaysia, for example, 15% of the population will be over 60 years old by 2030.^3^ Neurological disorders in older individuals reduce resilience, increase physical impairments in already frail individuals, foster dependency, have adverse impacts on psychological health and contribute substantially to increased care costs.^4^ Notably, epilepsy is more common as we age, increasing in incidence from 55 years, peaking after 75 years,^5-9^ with wide-ranging direct and indirect consequences.

Given the complex intersections between epilepsy and other non-communicable chronic illnesses in older people, an integrated care approach, that includes services for medical, social and rehabilitative needs specific to older adults, is essential. The removal of societal stigma and a focus on primary prevention are recurring themes that necessitate cooperation between communities, legislators and healthcare professionals. International mandates such as the Madrid Plan 2002 and the United Nations (UN) Convention on the Rights of People with Disabilities 2006,^10^ advocate for comprehensive policy frameworks aimed at preventing disability through non-communicable disease control, enhancing disability management via community-based rehabilitation and guaranteeing universal access to long-term care.

If, however, we are to make meaningful differences in brain health of older people, we need to better understand how common neurological conditions interconnect in this age group. Recent lines of enquiry have indicated a bidirectional relationship between epilepsy and dementia.^11-17^ Epilepsy, especially in older individuals, is associated with cognitive impairments similar to difficulties experienced in Alzheimer’s disease (AD).^18^ As a corollary, individuals with AD have an increased risk of seizures and epilepsy.^6^ Studies in AD demonstrate that tau pathology specifically associates with decreased cognitive function of brain regions that show increased tau accumulation,^19^ suggesting that cognitive decline may be driven by tau-mediated neurodegenerative mechanisms.^20^ Tau-mediated mechanisms might, therefore, underpin cognitive deficits in epilepsy, AD and possibly other tau-associated neurodegenerative conditions.^21,22^

Tau protein regulation, including phosphorylation, plays an important role in maintaining microtubule stability during neurodevelopment and is relevant to synaptogenesis. At a molecular level, tau ‘hyperphosphorylation’ is thought to be a pathological hallmark in AD and phosphorylated tau has been observed in epileptogenic pathologies.^23-27^ How clear, though, is our understanding of how the underlying biochemical changes, including complex and numerous post-translation modifications (PTMs), of the tau protein associate with pathology?

In this article, we aim to address the following questions: how do changes in tau biochemical, structural and cellular properties contribute to the cognitive difficulties in epilepsy? Could an improved understanding of tau-related mechanisms in epilepsy lead to biomarkers that better predict which individuals with epilepsy may be at risk of cognitive decline? We explore these novel avenues by examining tau from an epilepsy, rather than dementia, perspective and offer novel insights into how tau, dementia and epilepsy intersect in older people. We discuss how carefully considered integration of biomarkers may be helpful to stratify older people with epilepsy to best manage cognitive difficulties seen in this cohort. Lastly, we consider recent and ongoing anti-tau clinical trials aimed at removing pathological accumulation of tau in neurodegenerative conditions and individuals with epilepsy. We highlight how it is essential that cognitive neurologists, gerontologists and epileptologists across the world work together closely to tackle the rapid increases in mind-brain health disorders of older people.

## Tau protein structure and biochemistry

Tau, a microtubule-associated protein, was initially implicated in neurodegenerative disease following the identification of aggregated and fibrillar forms of the protein alongside amyloid-β (Aβ) plaques in AD.^28^ Pathological tau aggregation is also important in the pathogenesis of other neurodegenerative diseases including frontotemporal dementia (FTD), progressive supranuclear palsy (PSP) and corticobasal degeneration (CBD)—collectively referred to as tauopathies.^29^ The exact pathological role remains an area of investigation; however, several reports have shown the accumulation of tau aggregates in the brains of aged healthy individuals.^30^

Given the early recognition in AD, opinion has fluctuated over the years on whether tau pathology is a secondary phenomenon that is downstream to Aβ deposition^31^ or central to disease process.^32^ Identifying mutations in microtubule-associated protein tau (*MAPT*) leading to FTD provided stronger evidence of a causal role in neurodegeneration.^33-35^

Ambiguity around the exact pathological role of tau is partly due to the complexity of the protein sequence, the biochemistry involved and the structural properties of monomer and fibrillar forms. Tau monomers are intrinsically disordered, whereas tau fibrils are highly ordered and rich in β-sheet secondary structure.^36,37^ Electron microscopy and biochemical characterization of tau aggregates in post-mortem tissue from brains of patients with tauopathies have consistently shown two common features: the accumulation of hyperphosphorylated tau and the enrichment of fibrillar forms of the protein.^38,39^ A limited set of antibodies targeting specific phosphorylated forms of the protein are currently employed to detect, quantify and characterize pathological tau species in human brains and preclinical models of tauopathies.^40^ One limitation is that these antibodies were originally developed to target specific AD-associated pathological aggregates and, as a consequence, the precise hyperphosphorylation patterns distinguishing physiological and pathological forms of tau across different tauopathies remain largely unknown. In the context of epilepsy, no studies have yet attempted to systematically map the proteoforms or hyperphosphorylation patterns of tau in the brains of individuals with different forms of the condition. A key unanswered question, therefore, is whether existing antibodies targeting hyperphosphorylated tau can accurately capture the full diversity of tau pathology or if there are distinct epilepsy-specific hyperphosphorylation patterns.

### Tau structure and function

The *MAPT* gene on chromosome 17q21 encodes for tau protein.^41^ In the human brain, six isoforms are generated by alternative RNA splicing of exons 2, 3 and 10.^42^ Alternative splicing of exon 10 and the encoded second microtubule-associated binding repeat (MTBR) leads to 3R and 4R tau isoforms.^43^ Two different N-terminal domains of 29 amino acids and their presence or absence results in either 0 N, 1 N or 2 N isoforms leading to: 0N3R, 1N3R, 2N3R, 0N4R, 1N4R and 2N4R. An additional ‘big tau’ isoform containing an additional 4a exon is found in the peripheral nervous system.^44^

Tau is expressed primarily in axons of neurons, although it is found in lower levels in other cells such as glia^45^ with multiple interaction sites including the N-terminal region, C-terminal region, MTBR and proline-rich region.^46^ The MTBR region is positively charged and binds with negatively charged microtubules (MT) while the proline-rich region is also involved in MT binding and regulation. Tau has direct interactions with MT including binding, stabilization and promotion of MT assembly,^47^ as well as indirect effects on MT such as protection against end-length fluctuations^48^ and establishing a ‘spacer clear zone’ around MTs in cells.^49^

### Post-translational modifications of tau

Tau protein undergoes extensive and different PTMs that determine function, structure, cellular properties and aggregation potential. These include phosphorylation, acetylation, deamination, methylation, demethylation, nitration, *O*- and *N*-linked glycosylation, ubiquitination, Small Ubiquitin-like Modifier (SUMOylation) and proteolytic cleavage (for more details see Limorenko *et al*.^40^). The net effects of these PTMs upon the functional or pathological effects of tau depend upon the sites involved, the type of modifications observed and the cumulative effect all of these have upon the tau structure, its interactions with other proteins and its potential to form aggregates.^40^ Most tau molecules undergo multiple PTMs at various regions of the protein sequence, with distinct modification patterns observed across different tauopathies.^50,51^ These unique PTM signatures may influence tau aggregation, toxicity and disease progression, highlighting the need for precise characterization of tau modifications in each pathological context. The sequence in which these modifications occur, their interplay, and the identification of disease-specific or stage-specific PTMs remain largely unknown.^40^ Nonetheless, these aspects have garnered increasing interest in recent years, particularly in the context of AD.^50^ It is noteworthy that there are no studies on the PTM pattern of soluble or aggregated tau in the brain or biological fluids of people with epilepsy, with most studies on the role of tau in epilepsy focusing on the use of selected antibodies against phosphorylated and pathological tau in AD brains.

Certain pathological conditions can promote structural or cofactor-induced conformational changes in tau protein. As such, tau may shift from a highly soluble ‘paperclip’ conformation to a ‘misfolded’ form; this comprises nucleation or aggregation competent, di- or oligomers that may form nuclei or ‘seeds’, and finally aggregates. PTMs are not static but may change as a function of time, cellular localization, disease state or brain region/cell type.^40,52^ Although our understanding of the role of PTMs in driving tau aggregation and pathology formation remains incomplete, hyperphosphorylation of tau is recognized as a shared hallmark of various neurological diseases, including epilepsy, traumatic brain injury (TBI) and dementia.

### Is the term ‘hyperphosphorylated tau pathology’ a misnomer?

Phosphorylation is a commonly reported PTM with 85 predicted phosphorylation sites in human tau.^51^ In the case of tau protein, phosphorylation occurs on Ser/Thr or Tyr residues catalysed by three main classes of protein kinases. First, proline-directed kinases phosphorylate Ser/Thr residues, such as cyclin dependent kinase 5 (CDK5), CDK2, glycogen synthase kinase 3 beta (GSK-3β), mitogen-activated protein kinases p38MAPK and extracellular signal-regulated kinase (ERK)/MAPK.^53,54^ Second, non-proline-directed kinases, such as tau-tubulin kinases (TTBK), phosphorylate Ser/Thr residues that are not followed by a proline. Tyrosine kinases such as Abl kinase or Fyn kinase, phosphorylate tau at Tyr residues.^55,56^ Conversely, dephosphorylation is performed by phosphatases with protein phosphatase 2A (PP2A) being a prominent enzyme that accounts for over 70% of total tau dephosphorylation activity.^57^

Tau undergoes phosphorylation during several natural processes such as development, hibernation and in response to hypothermia.^58-60^ Physiological tau phosphorylation is associated with decreased metabolic rates and cellular management of stress periods. In some instances, multisite tau phosphorylation and inhibition of phosphatases can promote normal MT assembly.^61^ Tau phosphorylation at Tyr18 can positively regulate the dynamic stability of MT and promote physiological axonal trafficking.^62^ Additionally, phosphorylation at certain sites, including Ser262 and Ser214, protects against aggregation.^63^ This reflects a dynamic relationship between kinases and phosphatases. The term ‘hyperphosphorylated tau pathology’ can therefore oversimplify the role of phosphorylation as it does not describe either the level or site of modification. The primary reason for this oversimplification is that the commonly used methods for detecting and mapping tau phosphorylation states in the brain and biological fluids rely heavily on the limited number of antibodies which target a few specific pathology-associated phosphorylation sites. While mass spectrometry is increasingly utilized to profile physiological and pathological tau in AD and various tauopathies,^40^ antibodies remain the predominant tools for monitoring changes in phosphorylation levels and states.

### Pathological tau phosphorylation and aggregation

Phosphorylation has been closely associated with pathology as hyperphosphorylated tau aggregates form neurofibrillary tangles which are, alongside Aβ plaques, a pathological hallmark of AD.^64^ Neurofibrillary tangles correlate directly with progressive cognitive deficits which highlight phosphorylated tau as a potential driver of pathology.^65^ Similar aggregated tau is found in pathological markers of other primary tauopathies such as glial tau inclusions in tufted astrocytes in PSP, astrocytic plaques in CBD and globose tangles in frontotemporal lobar degeneration (FTLD)-tau.^66-68^

Biochemically, certain phosphorylated tau forms have been associated with impaired MT binding and mislocalization of tau from axon to the cytosol.^69,70^ Multisite phosphorylation of tau protein may lead to conformational changes that increase susceptibility to misfolding and aggregation.^71^ Aggregated tau fibrils conform to well-defined beta-sheets that possess a prion-like seeding capacity and can spread to other neurons,^72^ which has been demonstrated experimentally by injecting tau fibrils into brains of transgenic mice.^73^ Conversely, seeds that are dephosphorylated by phosphatases or immunized against phosphorylation are less effective at inducing tau pathology.^74^ The site of phosphorylation is again important as specific phosphorylated tau residues including Ser262, and combined Thr231/Ser235 found in AD brain lysate enhance tau seeding efficiency while other sites, such as Ser198/Ser199/Ser202 and Ser400/Thr403/Ser404, seem linked to lower seeding efficiency.^75^

Overall, the propensity for phosphorylated tau forms to promote misfolding, seeding and aggregates seems to depend on the site(s) involved, other structural changes (mutations, PTMs), or cofactors facilitating or inhibiting misfolding and aggregation. A study introducing pseudophosphorylation sites within various domains of tau, through mutagenesis, mimicking the negative charges of phosphorylations, suggested that mutations at the N-terminal regions inhibit, whereas C-terminal mutations promote, tau aggregation.^76^ The middle portion of tau exhibited variable effects upon aggregation when negatively charged residues were introduced via mutagenesis.

### Epilepsy and other misfolded proteins

Whilst not the focus of this review, it is important to highlight that there is growing evidence that neurodegenerative diseases are often characterized by the co-occurrence of multiple proteinopathies, with deposits and inclusions composed of Aβ, tau, α-synuclein (aSyn) or transactive response DNA binding protein 43 (TDP-43) found in the brains of affected individuals. In some cases, the abundance, distribution and type of aggregates have been shown to correlate with clinical symptoms and disease progression.^77-80^ Furthermore, the co-occurrence of aSyn, Tau, Aβ and TDP-43 pathology is also common in the brains of aged individuals without diagnosis of any neurodegenerative diseases.^81,82^ Although a few studies have reported Aβ plaques, aSyn and TDP-43 pathologies in the brains of individuals with epilepsy, the number and scope of these investigations remain too limited to establish a causal association or clear link to clinical symptoms.

#### Epilepsy and amyloid-β

During continuous video-EEG monitoring, early amyloid precursor protein (APP) transgenic mice models show both hyperexcitability, with frequent epileptiform activity, and non-convulsive seizures.^83^ This, at the time unexpected, observation led to extensive investigation as to how Aβ may disturb excitatory and inhibitory circuits.^84^ Other transgenic models, including presenilin 1, demonstrate co-localization of epileptic discharges with Aβ plaques supporting potential epileptogenic properties.^85,86^ Also, Aβ clearance through passive immunization prevents seizures in an animal model of AD.^85^

Considering human studies, amyloid PET imaging has investigated the burden and brain distribution of Aβ in patients with different types of epilepsy. One study reported increased Aβ deposition in individuals with childhood-onset epilepsy, which appeared to be influenced by apolipoprotein ε4 (APOE ε4) allele status rather than the specific epilepsy type or activity.^87^ This increased amyloid burden in epilepsy showed association with cognitive impairment. Another investigation, however, suggested that individuals with AD and epilepsy may have less Aβ pathology in the cingulate gyrus and perform better in cognitive tests compared with AD-only patients.^88^ Studies on Aβ plaques have also reported variable findings. Previous work by Silva and colleagues,^89^ reported low prevalence of Aβ plaques in patients with chronic drug-resistant temporal lobe epilepsy (TLE), whereas Fonseca and colleagues^90^ reported marked Aβ pathology accumulation in the mesial temporal regions and ipsilateral anterior cingulate gyrus of adults with drug-resistant TLE. Mackenzie and Miller^91^ found an age-accelerated occurrence of senile Aβ plaques in about 10% of the epilepsy cases without any association with cognitive decline. Aβ pathology may be an important link between epilepsy, hyperexcitability and cognitive impairment although it is likely that spatial patterns of Aβ deposition are more influential than overall burden.^88^ Aβ and tau co-pathology is also a highly relevant consideration. A recent disease-modifying therapy trial in AD showed tau progression was slowed by anti-amyloid therapy whilst high tau burden reduced the therapeutic impact of Aβ clearance on cognition.^92^ Further studies are required to understand whether Aβ and tau independently contribute to hyperexcitable networks and cognitive decline or, more likely, how these two proteins interact as part of the multifactorial nature of epileptogenesis in neurodegenerative pathologies.

#### Epilepsy and alpha-synuclein

aSyn is a presynaptic protein^93^ that has been implicated in synaptic transmission and neuronal plasticity. Misfolded, aggregated and predominantly fibrillar aggregated forms of aSyn comprise the neuritic and cytoplasmic inclusions known as Lewy bodies and Lewy neurites, which are key diagnostic hallmarks of Parkinson’s disease (PD) and other neurodegenerative diseases, including dementia with Lewy bodies (DLB) and multiple system atrophy (MSA), that are collectively referred to as synucleinopathies.^94^ aSyn-aggregate-specific PET tracers remain under development and thus there are limited brain imaging clinical studies on the contributions of aSyn pathology to the development, progression or clinical symptoms of epilepsy. Changes in aSyn expression or levels in biological fluids, serum and CSF, have been reported in different types of epilepsy^95-97^ and in some cases an increase in aSyn levels has been linked to disease severity,^98,99^ suggesting that levels of aSyn or aggregated forms of the protein in biological fluids may serve as a potential epilepsy prognostic biomarker.^100^ A more comprehensive review on the relationship between aSyn and epilepsy is provided by Ali and colleagues.^101^

#### Epilepsy and TDP-43

TDP-43 localizes in the nucleus of cells and shuttles to and from the cytoplasm to perform cellular functions.^102^ The misfolding and accumulation of TDP-43 within cytoplasmic inclusions is a defining hallmark of several neurodegenerative diseases that are commonly referred to as TDP-43 proteinopathies.^103^ The occurrence and role of TDP-43 pathology in people with epilepsy remain largely unexplored. Toscano and colleagues^21^ investigated the expression of TDP-43, phosphorylated tau and Aβ pathology in individuals with TLE and found no evidence of TDP-43 pathology or mislocalization in the brains of these patients.

Despite the common occurrence of Tau, aSyn and TDP-43 pathology in AD, PD and other neurodegenerative diseases, only one study has simultaneously assessed the burden and brain distribution of co-pathologies in AD with epilepsy cohorts.^104^ An observational study by Rocca and colleagues^105^ examined tau, aSyn and TDP-43 pathology in AD cases with and without epilepsy, and reported increased TDP-43 pathology in the middle temporal gyrus of AD patients with epilepsy. Further studies in larger cohorts are needed to clarify the relationship between neurodegenerative disease-related pathologies and the development or severity of epilepsy. It is also vital to determine whether the biology of epilepsy itself contributes to the emergence of these pathologies.

### Future questions around tau biochemistry

Several fundamental questions on tau PTM and aggregation remain unanswered, including: what are the primary molecular and cellular triggers and determinants of tau misfolding and aggregation? Which PTMs are protective and which are drivers of aggregation and neurodegeneration? Are there disease-stage and disease-specific PTM patterns? Which forms of tau are pathogenic and are there cell-type or disease-specific toxic forms of the protein? What is the role of the crosstalk between different tau PTMs in regulating its aggregation, clearance and toxicity? Our ability to answer these questions is limited by: (i) lack of understanding of the temporal relationship between the different tau PTMs; (ii) incomplete knowledge of which enzymes are involved in regulating aSYN PTMs; (iii) the use of cellular and *in vivo* modelling approaches that focus on investigating PTMs one modification at a time; and (iv) limited understanding of the biophysical and toxic properties of native (brain-derived) tau oligomers and non-fibrillar aggregates.^40^ Recent advances in cryo-electron microscopy have provided unprecedented insights into the structural properties of pathological tau filaments in AD and various tauopathies. These studies have revealed distinct, disease-specific fibril folds, underscoring the potential for structural classification of tauopathies.^106,107^ Thus far, though, there are no reports of studies aimed at deciphering the biochemical and structural properties of tau fibrillar or non-fibrillar aggregates from brains of preclinical models of epilepsy or people with epilepsy.

## Tau and experimental epilepsy models

The initial description of tau protein function in cytoskeletal MT binding has greatly expanded to encompass the dynamics of other intracellular organelles, including the axon initial segment,^108^ nuclear and axonal transport, mitochondrial output and presynaptic exocytosis. While derangement of the cytoplasmic tau interactome engages relatively slow processes leading to neurodegeneration,^109,110^ the presynaptic release of truncated tau also affects adjacent cells.^111^ Healthy tau expression mediates fast activity-dependent targeting of synaptic vesicle proteins and postsynaptic clustering of N-methyl-D-aspartate (NMDA) excitatory glutamate receptors.^112^ Mutation or pathogenic PTMs therefore control a complex and potentially unstable landscape mediating both rapid and progressive neurological deficits in the epileptic brain.^113^

### ‘Bad’ tau

In a healthy brain, dephosphorylated and phosphorylated tau exist in a tightly controlled homeostasis mediated by kinase and phosphatase (especially PP2A) enzymes. This balance is disturbed in conditions associated with seizures, including neurodegenerative conditions and TBI, leading to an accumulation of phosphorylated tau isoforms. Overexpression of pathogenic phosphorylated tau alleles increases epilepsy risk in AD, PD, PSP, FTD and after TBI via multiple mechanisms.^114^ Loss of tau alters voltage-gated potassium and sodium currents both in human stem cells^115^ and mouse brain.^116^ Neurons co-cultured with astrocytes expressing pathogenic tau isoforms exhibit hypersynchrony and hyperexcitability.^117^ In the rodent adult brain, selective interneuron accumulation of phosphorylated human tau by adeno-associated virus (AAV) overexpression suppresses adult hippocampal neurogenesis, stimulates local astrogliosis and disinhibits GABAergic transmission.^118^ The toxic effects of defective tau have also been widely studied in *Caenorhabditis elegans*, zebrafish and *Drosophila* models.^119^ Apart for tau hypomorphs in the shaker fly model,^120^ excitability phenotypes in these models have not been widely explored.

Abnormal P-tau has been demonstrated in various rodent models of TBI with associated epilepsy, including the controlled cortical impact model and in models of repetitive TBI,^121-126^ as well as in a zebrafish model.^127^ In the lateral fluid percussion injury (LFPI) rat model, where post-traumatic epilepsy is found in 22% of the rats,^128^ abnormal P-tau expression has been reported to follow different temporospatial patterns for each of the various P-tau forms.^52^ The AT8-specific staining (P-tau at Ser^129^/Thr^130^) shows an acute/subacute reaction pattern, being prominent 2 days after LFPI, whereas the AT180-specific staining (P-tau at Thr^125^) is more sustained between 2 days and 8 weeks post-TBI, in the ipsilateral cortical regions.^52^ There is a complex pattern of changes in the various PTMs of tau after TBI, and the exact role they play in post-TBI pathologies is currently unclear.

Conversely, seizures might contribute to abnormal tau pathologies. In the zebrafish TBI model, TBI triggered seizures and tau aggregates (4R) in the brain. Seizures induced by exposure to kainic acid (but not 4-aminopyridine) further worsened the observed tauopathy.^127^ Treatment with retigabine, an anti-seizure medication, reduced the tau aggregates. In the rat LFPI model, low plasma tau levels (detected with the AT180 antibody) differentiated the LFPI rats from controls [receiver operating characteristic area under the curve (ROC AUC) = 0.875] and this was a strong peripheral biomarker of early post-traumatic seizures in vehicle-treated LFPI rats (ROC AUC = 1). It is unclear what drives the low plasma P-tau levels in the setting of TBI and post-traumatic seizures. This may, for example, reflect sequestration towards the brain or altered antibody sensitivity for the plasma tau forms present in these settings. Prophylactic levetiracetam treatment, starting immediately after the injury, reverted plasma P-tau levels to control levels, even though it did not abolish early seizures.^131^

### Better to have no tau than ‘bad’ tau?

Several studies have examined the effect of genomically deleting *Mapt1* in wild-type mice and mouse disease models. Wild-type mice with congenital^132^ and adult-onset^133^ lack of tau are remarkably free of neuropathology and neurocognitive phenotypes. Deletion of tau dramatically suppresses epilepsy and premature mortality in monogenic seizure models. Tau deletion spared the neurodegenerative effects of Aβ/Fyn and reduced the hyperexcitability and seizures seen in transgenic murine models over-expressing human APP.^134^

In a ‘pure epilepsy’ K_v_1.1 deletion model, absence of tau protein suppressed epilepsy as well as its downstream effects of megalencephaly and premature mortality.^120^ This study also reported the prevention of the hyperexcitability limb-shaking phenotype in shaker *Drosophila*. A similar effect was seen in the *SCN1A* Dravet syndrome mouse model.^135^ These studies showed that tau ‘dosage’ was important since heterozygous knockout animals exhibited an intermediate level of seizures and life span. Subsequent work has revealed that conditional deletion of tau in excitatory neurons alone can prevent seizures in the *SCN1A* mouse model, while loss of tau in interneurons had little effect.^136^ Also, tau deletion suppresses autistic features in the *FMR1* mouse model^137^ although congenital loss of tau is not effective in all channelopathy epilepsy models.^138^

## Tau and human epilepsy: pathological evidence

Recent studies of tau protein in tissues from people with drug-resistant epilepsy have raised the possibility that seizures can accelerate neurodegenerative processes. We still need to understand whether the tau topography is unique in epilepsy or conforms with stages of well-characterized tauopathies, e.g. AD, PSP, FTD, chronic traumatic encephalopathy (CTE), primary age-related tauopathy (PART) or age-related tau astrogliopathy (ARTAG), in addition to exploring tau biochemistry and important co-pathology. The importance of any relationship between tau accumulation and memory/cognitive impairment, as well as cortical atrophy on neuroimaging, will be relevant to establishing tau’s significance as a disease biomarker for progressive neurodegeneration in epilepsy.

To date, published series have shown somewhat variable findings (Table 1). These investigations consist of heterogenous studies that span different clinical cohorts. Here, we have not applied a formal quality assessment tool as such and instead provide a critical analysis of available evidence. There appears to be a generally lower total ‘tau load’ in epilepsy compared with well-studied, established secondary tauopathies such as AD (Table 1).^21,23,25,89,139,141-147,149^ Studies conducted in epilepsy surgical cohorts are, by their nature, cross-sectional, limited to the resected brain region and often based on small samples in mainly younger age groups as opposed to post-mortem neurodegenerative disease studies. Whilst such work is unable to address disease staging or progression, it provides some evidence that older epilepsy cohorts, often with longer durations of epilepsy, show greater overall tau burden than might be seen with an age-dependent accumulation (Table 1).

**Table 1:** Recent pathology studies of tau in epilepsy surgical resections including types of tau studied, brain regions included, pathology type and the proportion of cases with tau identified in relation to age group at surgery

Study	Cases, *n*	Pathology or focal epilepsy; region studied	Tau types	Age groups (years) and P-tau cases (%)	Other proteins examined	Cognitive correlation with tau	Tau pattern
Toscano *et al*.^21^	22	HS type 1/2; hipp	CP13 (Ser 202)	30–58 years: 95%	Aβ, TDP-43 all negative	Attention deficit	Glial deposits (95%)
*Tai et al*.^139^	33	TLE-HS; temporal + hipp	AT8	50–65 years: 93%	Aβ 15%	Verbal decline postoperatively	Superficial, subpial, mossy fibres
Sen *et al*.^23^	15	FCD II; frontal/temporal/parietal	AT8, 3R/4R	1–81 years: 73%	Aβ 13%	–	Dysplastic region
Hwang *et al*.^140^	11	TS; ACC, IFG, EC, hipp	CP13, PHF1 (Ser 396–404), 3R/4R,Tau Ac (K274, K343),	30–58 years: 63%	Aβ, TDP-43 all negative	–	Ac > P-tau; 3R > 4R non-Braak distribution
Aroor *et al*.^141^	12	HS, FCD I/III; lat temporal	AT8 (Ser 202, Thr205), Thr 205,Thr161	24–67 years: 50%	Aβ 1%	IQ, no correlation	pS6 correlation
Liu *et al*.^142^	10	TS; DLPFC, IFG, EC	AT8, GT38, AV1451	52 years (mean): 50%	Aβ all negative	–	3R > 4R
Iyer *et al*.^143^	36	FCD II, TS; frontal/temporal	AT8	18–51 years: 42%	Abeta all negative	–	Co-localized pS6
Smith *et al*.^144^	60	HS/non-lesion; temp/frontal/parietal	AT8	19–45 years: 38%	–	–	–
Kakita *et al*.^145^	108	FCD I/II/non-les; frontal/temp/etc.	AT8	4–61 years: 27%	Not assessed	–	NR
Jones *et al*.^146^	10	TLE/FLE; Frontal, temporal	AT8	18–45 years: 10%	–	–	–
Silva *et al*.^89^	56	HS/path-neg; lat temporal + hipp	AT8	20–68 years: 3%	Aβ 7%	No association	Not described
Prada Jardim *et al*.^147^	92	TLE-HS; hipp	AT8	18–55 years: 1.3%	–	Postoperative decline	–
Putra *et al*.^148^	6	TLE/FLE; lat temporal (4), R frontal (2)	AT8, Y18 (tyrosine kinase)	24–72 years: NS	Increase versus controls	–	PLA Fyn; Tau also increased compared with controls
Puvenna *et al*.^25^	19	TLE/FLE/OCC; temporal cortex	AT8, CP13, Tau5 (total tau), insoluble tau	1–58 years: NS	–	–	Superficial/pial/perivascular
Gourmaud *et al*.^149^	19	TLE-HS/FCD/DNT; temporal + hipp	AT8, AT180 (Thr 231), 4R, TAU5	10–56 years: NS	APP, Aβ42	Executive function deficit	AT180 in glial cells

Studies are listed in descending order of P-tau percentage. Ac = acetylated tau; ACC = anterior cingulate cortex; DLPFC = dorsolateral prefrontal cortex; DNT = dysembryoplastic neuroepithelial tumour; EC = entorhinal cortex; FCD = focal cortical dysplasia; FLE = frontal lobe epilepsy; hipp = hippocampus/hippocampal; HS = hippocampal sclerosis; IFG = inferior frontal gyrus; lat = lateral; NR = not reported; NS = not specified; OCC = occipital lobe epilepsy; P-tau = phosphorylated tau; TLE = temporal lobe epilepsy; TS = tuberous sclerosis.

Tau accumulation has been studied in acquired pathologies such as TLE with hippocampal sclerosis (TLE-HS), developmental lesions and glioneuronal tumours in individuals with symptomatic epilepsies. In TLE-HS, there can be preferential phosphorylated tau accumulation in the neocortex over limbic regions with involvement of superficial cortical neurons in addition to sprouted axons.^25,89,139^ This pattern differs from conventional Braak staging in AD and PART, where tau accumulation initiates in limbic structures before spreading to neocortical areas.

In the adult brain, tau phosphorylation is tightly regulated and induced by NMDA receptor activation.^150^ The kainic acid model of status epilepticus, however, shows dynamic tau phosphorylation in mossy fibre axons of the hippocampus following seizures.^151^ Observed tau accumulation in TLE may reflect disease activity or seizure networks in the epileptogenic region and, moreover, may be transient and reversible. This distribution of tau in TLE is also similar to the superficial gyral cortical tau accumulation described in Nodding syndrome, a severe endemic form of atonic epilepsy with degeneration, likely secondary to *Onchocerca volvulus* infection.^152^

Phosphorylation of certain tau sites occur as early events relevant to the initiation of tau oligomerization, further conformational change and cellular aggregation. Phosphorylation and other PTMs may also occur after tau aggregation and play important roles in regulating tau pathology maturation, toxicity, interactome or clearance. Phosphorylation at specific tau sites, though, may be reversible or protective.^51,140^ Studies in epilepsy have not yet fully addressed conformational forms of tau and have mainly confirmed similar phosphorylation of tau on sites recognized in AD (Table 1). Tangles, pre-tangles and insoluble tau forming primarily in neuronal, rather than glial, populations have also been described.^25^ In subacute sclerosing panencephalitis, a condition typically accompanied by seizures where tau accumulates in the superficial cortical neurons, cryogenic electron microscopy showed an identical tau filament structure to CTE.^153^

**Figure 1:**
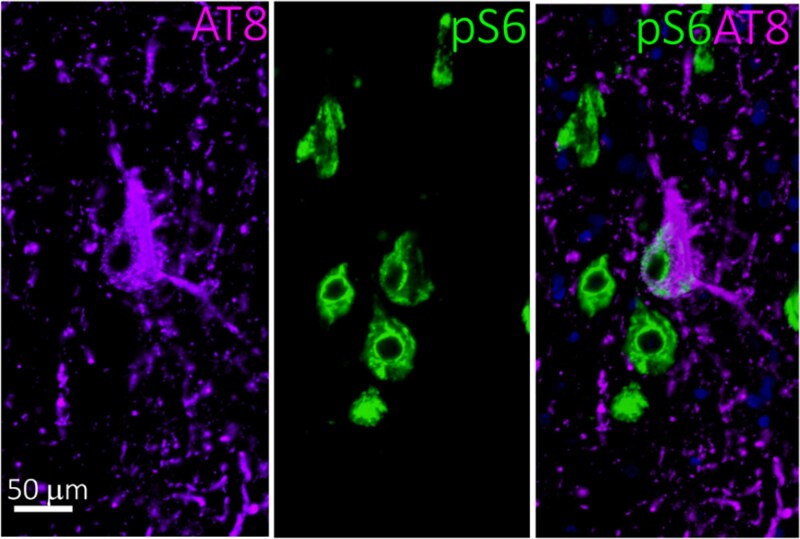
**Focal cortical dysplasia type IIB with dysmorphic neurons, some of which showed P-tau with AT8 immunohistochemistry.** Double labelling with pS6 immunohistochemistry (Ser240/244) for mTOR pathway activation confirmed upregulation in neurons including cells with AT8 expression. Image Courtesy of Alicja Mrzyglod, University College London, UK.

In the developing brain, the 3R tau isoform predominates with phosphorylation of tau at pSer214, pSer202, pSer396/Ser404, observed particularly in the marginal zone in the later stages of development.^154^ In a tauopathy recently described in older people with tuberous sclerosis complex (TSC), 3R tau was the dominant form,^155^ although others have found both 3R and 4R isoforms.^142^ Phosphorylated tau accumulation in dysmorphic neurons in focal cortical dysplasia type II and glioneuronal tumours has also been linked with neuronal mammalian target of rapamycin (mTOR) pathway activation, which may be more widely relevant in focal epilepsy as demonstrated by concurrent labelling for pS6 (Fig. 1).^141,143,156^

In post-mortem studies, examination of the brains of people with TSC, a non-Braak distribution of tau was shown, suggesting different mechanisms may be driving tau accumulation and disease propagation in the mToropathies.^155^ Also, in many epilepsy-associated pathologies tau accumulation does not occur in parallel with, and likely appears independent of, significant Aβ or other protein accumulation (Table 1). In a post-mortem epilepsy series with mixed aetiologies, higher Braak stage and astrocytic tau protein were associated with TBI. There was no clear association between seizure types, frequency of seizures, age at onset or duration of epilepsy.^24^ In the same study, higher Braak stages were found more commonly in the focal onset epilepsies than in the generalized epilepsies. In a more recent post-mortem study, using the Corsellis Epilepsy Collection, CTE neuropathological changes were seen in 15.7% of epilepsy post-mortem brains. These patients exhibited a higher rate of memory impairment (53%), sudden unexpected death, younger age of epilepsy onset, TBI history and institutionalization compared with CTE. Again, no association with seizures was seen.^157,158^

On review of the variability in findings examining tau pathology in epilepsy, several factors must be considered across different studies including differences in methods used, brain regions sampled and populations studied. Resected hippocampi tend to show less proportional tau positivity^89,139,144,147^ with the epileptogenic neocortex showing higher tau positivity.^89,139,144^ The opposite effect has, however, also been observed—namely greater proportional burden in the hippocampus^21^ but not the temporal neocortex.^141^ Differences in cohort age and the brain regions investigated affect the interpretation of results in TSC. Moderately increased P-tau is observed in the frontal and temporal regions in a subset of patients,^155^ and in dysplastic brain regions in people older than 30 years. Another potential confounder is use of semi-quantitative tau scales with variable cut-offs for being labelled ‘positive’. Greater consistency of results is seen in focal cortical dysplasia, wherein tau burden is present in the dysplastic regions of older patients (>40 years) but not the surrounding normal cortex.^23,143^

In summary, there is growing evidence of tau pathology in people with epilepsy although heterogeneity exists along several dimensions including age, underlying pathology, epilepsy characteristics, previous TBI as well as genomics. Going forward, quantification methods, the tissue sampled and immunohistological methods need to be standardized to enable accurate investigation of tau pathology in different epilepsy subtypes. This will lead to a more exact understanding on the biochemical, structural, aggregation and toxic properties of tau in epilepsy.

## How do tau, epilepsy and neuropsychological testing interrelate?

Investigating neuropsychological performance in relation to tau pathology in resected epileptogenic tissue has yielded important, albeit inconsistent, clinico-pathological insights.^159^ Studies examining tissue from patients with TLE report varying degrees of hyperphosphorylated tau burden ranging from 3.5% to 95% and Aβ deposits ranging from 7% to 67% (Table 2). One study found that higher tau burden in temporal lobe tissue was associated with greater postoperative declines in verbal learning, recall and naming abilities—despite all patients becoming seizure-free following the resection.^139^ Comparatively less tau was found in the hippocampus, which may relate to extensive neuronal loss in this region. Others have also found increased levels of phosphorylated tau in resected hippocampal tissue from patients with TLE^21,149^ correlating with impairments in verbal and visual memory, memory span, language, attention and executive function. Tau burden was associated with impaired attention and higher seizure frequency,^21^ as well as impaired preoperative executive function.^149^ These findings suggested tau pathology may contribute to cognitive deficits in some people with epilepsy, via mechanisms similar to those seen in tau-related neurodegenerative diseases.

**Table 2:** Studies examining P-tau burden in temporal lobe resections in relation to cognition

Reference	*n*	Age, years (range)	P-tau and amyloid	Controls	Results
Tai *et al*.^139^	33	53.6 (50–65)	P-tau 94% Aβ plaques in 15%	Age-matched population controls from a post-mortem series	No significant association between P-tau and presurgical neuropsychological performance. A higher P-tau burden was inversely correlated with cognitive decline from pre- to 12 months postoperative [verbal learning (*r* = −0.63) and memory (*r* = −0.44) and naming (*r* = −0.50)] as well as from 3 months to 12 months postoperative [verbal learning (*r* = −0.54)].
Gourmaud *et al*.^149^	19	29 (10–56)	Tau: not available APP in 3/11 = 27%	Twenty-two neurologically normal and nine AD autopsy cases	Both the total and P-tau burden inversely correlated with presurgical deficits in executive functions, including processing speed efficiency (*r* = −0.78) and verbal working memory (*r* = −0.89).
Silva *et al*.^89^	56	34 (20–68)	P-tau 3.5% Aβ plaques 7%	–	No significant correlation between P-tau and presurgical memory performance was found.
Toscano *et al*.^21^	22	42 (30–58)	P-tau 95% No Aβ deposits	Twenty hippocampi of neurologically normal autopsy cases	Higher P-tau burden associated with deficits in confrontative naming and verbal memory span.
Aroor *et al*.^141^	12	43 (24–67)	P-tau 50% Aβ deposits in 67%	–	No significant correlation between P-tau levels at two different sites (Thr181 and Thr205) and intelligence was found. The non-significant positive (!) correlation coefficient was large at site Thr181 (*r* = 0.54; *P* = 0.16).

By contrast, other analyses of resected temporal lobe tissue have revealed either substantial tau pathology without significant correlation to full-scale intelligence quotient (IQ)^89^ or low P-tau burden with no link to pre- or postoperative cognitive performance.^141^ The authors of these studies argued against AD pathology as a primary driver of cognitive deficits in epilepsy, though they acknowledged that ageing might interact with tau accumulation to shape cognitive outcomes.

Beyond resection analysis, Fernandes and colleagues^160^ examined CSF tau levels in a cohort of people with later-onset epilepsy of uncertain origin (LOEU) finding that both total and P-tau levels were elevated with associated lower cognitive performance than controls. The authors pointed out the similarities in CSF biomarker patterns in both LOEU and AD. This led to the hypothesis that LOEU might, in some cases, represent an undiagnosed or prodromal form of AD.

Taken together, it remains uncertain whether tau accumulation plays a causal role in the cognitive deficits observed in epilepsy. Age-related effects appear particularly relevant, with studies reporting stronger associations between tau and cognition in older cohorts.^139,160^ One possibility is that chronic seizures or related factors (e.g. inflammation, excitotoxicity) accelerate brain ageing and tau accumulation. Another key question is the role of additionally present Aβ pathology. It may be that another biomarker of neurodegeneration was present at different levels across analyses and it remains open how it relates to P-tau and cognitive performance in epilepsy.

Causal inference must therefore be approached cautiously, particularly within the framework of the proposed ‘tri-directional’ relationship between epilepsy, cognitive decline and neurodegeneration.^15^ Numerous factors—such as seizure frequency, anti-seizure medication effects, psychiatric comorbidities and structural brain damage—contribute to cognitive outcomes and may interact with tau pathology.^161-163^ Moreover, discrepancies in study methodologies, age ranges and neuropsychological instruments further complicate interpretation. To assess the contribution of tau to cognition more reliably, future studies should incorporate broad age spectra, apply sensitive and standardized cognitive testing,^164,165^ and include longitudinal designs that account for epilepsy-specific factors such as seizure severity, duration and injury history.

## Neurodegenerative proteinopathies and seizure prevalence

While epilepsy is associated with increased tau pathology, people with neurodegenerative proteinopathies have an increased risk of developing epilepsy. A retrospective study of 2054 patients with neurodegenerative dementia reported a 1.85% epilepsy rate, with seizure onset around or after the clinical diagnosis of dementia.^166^ In this cohort, seizure prevalence ranged from 1.28% in behavioural variant FTD (bvFTD), over 1.82% in AD, 2.47% in DLB to 12% in primary progressive aphasia (PPA). In comparison, the overall prevalence of epilepsy in older populations ranges between 0.5% and 0.89%.^167^

The increased prevalence of epilepsy in neurodegenerative disorders may relate to pro-epileptogenic properties of the pathological protein, the neurodegenerative process itself or the location of pathology.^114,168^ Most studies have been conducted in AD, where seizures may precede and accelerate cognitive decline.^169,170^ Seizure prevalence in primary tauopathies (FTD, PSP, CBD) and synucleinopathies (PD, DLB, MSA) are less well studied. A large UK retrospective cohort study using primary care data identified that seizure incidence in PD was slightly increased (incident rate ratio of 2.37) compared with controls (Table 3).^171^

**Table 3:** Prevalence of seizures in different neurodegenerative proteinopathies based on clinical diagnoses and/or neuropathological diagnoses

Protein	Clinical Dx	Sample (*n*)	Prevalence (%)	Observation period (years, median)	Neuropathological diagnosis	Sample (*n*)	Prevalence (%)	Disease duration (years, mean)
Amyloid-β; tau	AD^167^	1320	4.6	5.2	AD^169^	144	31.3	10.9
	AD^161^	1645	1.8	NA	AD^170^	4509	7.8	NA
	AD^168^	1326	1.4	10 years pre Dx	–	–	–	–
			5.0	at Dx	–	–	–	–
			6.9	5 years post Dx	–	–	–	–
Alpha-synuclein	PD^166^	23 086	1.2	21.0^a^	–	–	–	–
	DLB^167^	149	2.0	5.5	LBD^169^	103	12.6	13.6
	DLB-AD^167^	29	20.7	5.5	LBD^170^	413	6.3	NA
	DLB (2)^161^	81	2.5	NA	–	–	–	–
	–	–	–	–	LBD^169^	103	12.6	13.6
Tau	PSP^167^	54	1.9	5.5	PSP^169^	93	7.5	8.2
	CBS^167^	61	3.3	5.5	CBD^169^	25	20.0	6.6
	bvFTD^167^	115	3.5	5.5	FTLD-Tau^169^	14	28.6	6
	bvFTD^161^	235	1.3	NA	FTLD^170^	745	4.8	NA
	nfvPPA^167^	37	5.4	5.5	–	–	–	–
	PPA^161^	25	12.0	NA	–	–	–	–
	FTD^168^	245	3.3	10 years pre Dx	–	–	–	–
			6.5	at Dx	–	–	–	–
			11.2	5 years post Dx	–	–	–	–
	–	–	–	–	AGD^172^	7	57.1	0.5–3
TDP-43	svPPA^167^	82	0	5.5	FTLD-TDP^169^	39	5.1	6

AD = Alzheimer’s disease; AGD = argyrophilic grain disease; bvFTD = behavioural variant; CBD = corticobasal degeneration; CBS = corticobasal syndrome; DLB = dementia with Lewy bodies; DLB-AD = dementia with mixed DLB and AD pathology; Dx = diagnosis; FTD = frontotemporal dementia; FTLD = frontotemporal lobar degeneration; LBD = Lewy body disease (comprising DLB and PD); MSA = multi-system atrophy; NA = not available; nfvPPA = non-fluent variant PPA; PD = Parkinson’s disease; PPA = primary progressive aphasia; PSP = progressive supranuclear palsy; svPPA = semantic variant PPA.

^a^Refers to maximal observation period.

An observational study over 6 years in 1846 patients with AD, DLB or FTD/PSP identified new-onset seizures in 4.5%, 5.1% and 2.6%, respectively, with incident rate ratios of 9.7 (AD), 10.0 (DLB) and 6.4 (FTD/PSP).^173^ Age-stratified incidence rates showed high seizure occurrence in young AD individuals (<50 years), older people with DLB (>70 years) and particularly in individuals with DLB and concomitant AD characteristics.^173^ In the tauopathies, seizure incidence was highest in the non-fluent PPA (nfvPPA), followed by bvFTD, PSP and corticobasal syndrome (CBS), whilst no seizures were reported in semantic variant PPA (svPPA) individuals (Table 3). A recent longitudinal case-control study in patients with AD (*n* = 1326) and FTD (*n* = 245; no subtype specification) compared the prevalence of epilepsy to age-matched controls (*n* = 2416) at three time points.^174^ Prevalence was higher in FTD than in AD and healthy controls at diagnosis (6.5%, 5.0%, 1.8%, respectively); 5 years thereafter (11.2%, 6.9%, 2.2%, respectively) and 10 years before diagnosis (3.3%, 1.4%, 0.8%, respectively), which supports the hypothesis of a specific role of tau in epileptogenesis.

By contrast, seizure rates were substantially higher in a series of 454 patients with neuropathologically proven AD (31.3%), CBD (20.0%), DLB (12.6%), FTD (11.3%), MSA (8.3%) and PSP 7.5% (Table 3).^168^ When comparing FTD subtypes, people who have FTLD with TDP43-immunoreactive pathology (FTLD-TDP) had lower seizure prevalence compared with FTD-tau patients.^168^ Analysis of the first clinical symptoms in the same cohort revealed only one PSP patient with a ‘convulsive attack’.^175^ Importantly, the mean age of developing cognitive symptoms was significantly associated with increased seizure prevalence. The high seizure prevalence reported in this neuropathological study may be attributable to the long observation period, from diagnosis to very late disease stages. A recent multicentre study in 6085 autopsy-proven dementia cases found that active seizures were associated with a higher load of AD-pathology across all dementias studied,^176^ although there were overall lower seizure rates in AD (7.8%), DLB (6.2%) and FTLD (4.8%) than other cohorts.

TDP-43 protein is also associated with an AD-like syndrome which was recently named limbic-predominant age-related TDP-43 encephalopathy neuropathologic change (LATE-NC).^177^ Study of post-mortem cohorts identified LATE-NC in a third of individuals above the age of 85 years.^172^ Independent of other proteinopathic changes, LATE-NC is associated with ante-mortem cognitive deficit as well as hippocampal sclerosis.^178-180^ The relationship between TDP-43 and seizures has been poorly examined. One recent brain bank investigation identified greater incidence of TDP-43 within the medial temporal gyrus in cases of AD plus epilepsy compared with AD without epilepsy.^181^

In another neuropathological series, 7 of 55 patients aged below 75 years were identified at post-mortem with primary tauopathy argyrophilic grain disease without significant concomitant pathologies.^182^ Four of these seven had seizures and pathological EEG findings.

Seizure prevalence, therefore, appears higher in neurodegenerative proteinopathies involving mesio-temporal or cortical structures, such as AD, DLB, CBD, bvFTD and to a lesser extent in disorders mainly affecting subcortical structures, such as PD, MSA and PSP. Notably, svPPA associated with TDP-43 has a lower seizure prevalence than nfvPPA, a primary 4R-tauopathy, despite both disorders affecting cortical structures. This potentially further supports a specific role for tau in epileptogenesis.

## Can we use tau as a biomarker for cognitive decline in people with epilepsy?

### Imaging biomarkers

AD research has benefitted from the availability of novel PET tracers identifying neurodegenerative deposits such as Aβ pathology and, more recently, tau.^51^ In AD, tau is considered the most relevant biomarker for monitoring disease progression. Unlike Aβ load, tau levels closely correlate with cognitive decline and tau PET tracer measurement aligns well with the spread of AD pathology. While current tau PET can assess tau burden and location over time, the first generation of tau PET tracers only partially depicted tau load in AD leading to several challenges around early detection and prognostication of disease.^183-185^

Owing to the urgent clinical need for *in vivo* tau measurement, translation from preclinical models and post-mortem histopathology-PET correlation to human tau PET tracer studies have occurred at a substantial pace. Tau PET tracer ^18^F-AV-1451 is approved by the Food and Drug Administration (FDA) for imaging tau in AD but has limited utility in measuring non-AD tau.^186,187^ Other first-generation tracers, such as THK-5351, were found to have significant off-target binding and their use has been discontinued as tau imaging agents. Instead, some of these tracers are in development for astrocyte imaging due to high levels of binding to monoamine oxidase-B (MAO-B).^188,189^

Second- and third-generation tracers such as ^18^F-PI2620, ^18^F-MK6240 and ^18^F-APN-1607 have improved imaging characteristics, being more sensitive to tau accumulation and demonstrating improved specificity for tau.^189-195^ Of note, these newer tracers demonstrate specificity for AD (3R/4R tau) and non-AD related tau (4R tau) being able to differentiate cortical and subcortical accumulation of tau in different neurodegenerative diseases.^196,197^

Exact characterization of tracer binding to distinct types of tau aggregates, reflecting common disease phenotypes from prodromal (tau fibrils) to advanced stages (intracellular inclusions) are still being elucidated.^194,198^ This is important to guide the rational choice of tracers and to facilitate interpretation at a clinical level.

Notwithstanding these challenges, the use of PET in AD and ageing research contrasts with PET studies in epilepsy. Clinical studies in epilepsy have so far focused mainly on the mapping of glucose metabolism for presurgical purposes [i.e. through ^18^F-fluorodeoxyglucose (FDG)] rather than examining for neurodegenerative deposits. One recent study investigated tau deposition in patients with TLE using the *in vivo* PET tracer ^18^F-MK-6240.^199^ Tau-PET uptake was increased with greater impairment of episodic memory and executive function. Tau accumulation also increased with longer disease duration. This was most prominent in the contralateral temporal cortex demonstrating pathological accumulation of tau outside of the epileptogenic zone and matching certain pathological observations.^24^ Such data provide clear evidence for the use of tau PET in understanding neurodegeneration and cognitive decline in people with epilepsy. Nonetheless, a more comprehensive assessment and comparison of tau pathology in epileptogenic areas using an expanded toolbox of antibodies and mass spectrometry is essential to determine whether the existing tau tracers can detect pathological forms of epilepsy-associated tau aggregates.

### Fluid biomarkers

Fluid biomarkers of tau pathology are an emerging technique for detecting AD pathology. Once the domain of academic research, CSF and plasma measurements of P-tau have transitioned to feature in the recent diagnostic criteria for AD.^200^ The ratio between CSF T-tau and P-tau is thought to reflect the excess levels of P-tau that are secreted from neurons containing pathological neurofibrillary tangles, the aggregated form of hyperphosphorylated tau.^201^

Recent clinical studies have focused on measuring specific phosphorylated forms of tau including P-tau181, P-tau217 and P-tau231 with others in development.^202-204^ While P-tau181 was considered the ‘classic’ AD biomarker, recent findings suggest that P-tau217 may correlate more strongly with both tau PET measurements and with earlier Aβ PET changes.^205^ In addition, detection of brain-derived tau, i.e. small molecular weight tau fragments, in blood has shown promise as a biomarker of Aβ-associated neurodegeneration and cognitive decline in AD,^206^ correlates with CSF total tau,^207^ and functional outcomes after stroke^208^ or severe TBI.^209^

Fluid biomarkers of tau pathology and the advent of ultrasensitive immunoassays, such as electrochemiluminescence,^210^ have significant clinical potential. A recent longitudinal study of the Swedish Biomarkers for Identifying Neurodegenerative Disorders Early and Reliably (BioFINDER-2) cohort combined blood-based measurements of P-tau217 and Aβ42/40 to predict the future accumulation of amyloid PET and CSF levels of Aβ in individuals without cognitive impairment over a 5-year period,^211^ finding this to be more accurate than a dementia specialist.^212^ This approach may allow for the very early detection of a neurodegenerative AD process and potential use in primary prevention trials.

P-tau concentrations do not appear to reflect pathology to the same extent in other neurodegenerative tauopathies despite the pathological presence of tau in neuronal inclusions or in glial cells in conditions such as PSP and forms of FTD.^30^ P-tau levels in non-AD tauopathies are often not detectable or are similar to healthy controls in CSF or blood.^129^ The reasons for this mismatch of pathology and detection are not entirely clear, although the presence of Aβ pathology may be relevant in facilitating release of tau from neurons.^213^ This is a growing field with CSF P-tau231 recently being found in increased levels in non-AD tauopathy carriers of the MAPT mutation R406W.^214^ How this assay performs in sporadic non-AD tauopathies has yet to be determined. Increases in total tau reflect non-specific neurodegeneration and are observed in chronic neurodegenerative diseases as well as following acute injury associated with neurodegeneration such as stroke and brain trauma.^130,215^ How levels of T-tau and P-tau may correlate with seizures in neurodegenerative disorders, though, remains currently uncertain.

One possible explanation for the lack of measurable tau and P-tau in tauopathies is the intracellular sequestration of tau and differences between peripheral and BD-tau. Peripheral, ‘big tau’, represents a large proportion of detectable tau in blood, but the relatedness of these measures to central, ‘brain-derived’ tau is not necessarily linear. Recently, a specific antibody for brain-derived tau has been developed which demonstrates a linear correlation between CSF tau and blood-brain-derived tau.^206^ Notably, brain-derived tau is detectable in non-AD tauopathies, suggesting it may have broader applications. The correlations between CSF and blood-brain-derived tau are not, however, present in FTD,^216^ and therefore need careful evaluation when used outside of AD. These brain-derived tau measures have yet to be benchmarked against tau PET but could rapidly advance the ability to detect and track tau in the brain.

It should be emphasized that the PTM patterns of soluble tau from people with epilepsy has not been assessed. A recent study demonstrated that while tau isoforms in insoluble aggregates vary between tauopathies, their soluble counterparts remain similar. Distinct PTM signatures in both soluble and aggregated tau, such as ubiquitination at Lys-369 in CBD and acetylation at Lys-311 in Pick’s disease, provide potential targets for developing fluid-based biomarkers to distinguish tauopathies *in vivo*.

### Which neurodegenerative biomarker and when?

Choosing when to test a specific biomarker is an ongoing debate within the AD and neurodegeneration fields. Broadly, there is high sensitivity and specificity across key fluid and imaging biomarkers based on different source pathology and affected by factors such as the assay deployed and the cohort tested. Tau and amyloid PET imaging, validated with post-mortem histopathology, show high sensitivity and specificity (generally between 90% and 95% for both).^217,218^ One study identified a slightly higher sensitivity of amyloid PET compared with ^18^F-FDG-PET with similar specificity for intermediate level AD pathology.^219^

Plasma and CSF tau/Aβ biomarker studies have used ante-mortem imaging data for validation. P-tau biomarkers had greater diagnostic performance and larger effect sizes compared with total tau and Aβ42/40 assays in detecting Aβ pathology.^220^ In particular, plasma P-tau217 is now comparable to CSF and PET-based biomarkers for AD diagnosis with sensitivity and specificity between 85% and 95%—a major diagnostic advance.^221^ A prominent strategy for suspected AD proposes using a two-cut-off methodology to group individuals into low, intermediate and high levels of P-tau217. This approach increased the diagnostic accuracy in a diverse cohort of individuals with cognitive impairment across several countries.^222^

Whether findings from the AD field will translate directly to individuals with epilepsy remains untested. Also, plasma markers of tau and Aβ are not consistently elevated with other tauopathies. In those conditions tau biomarkers may not be the most informative test.^30,129^ Clinical acumen remains critical. For example, in instances of atypical features or parkinsonism, plasma neurofilament light chain (NfL), as a measure of active neuronal damage and degeneration,^223^ as well as more general functional imaging such as FDG-PEG may offer better utility.^224^

**Figure 2:**
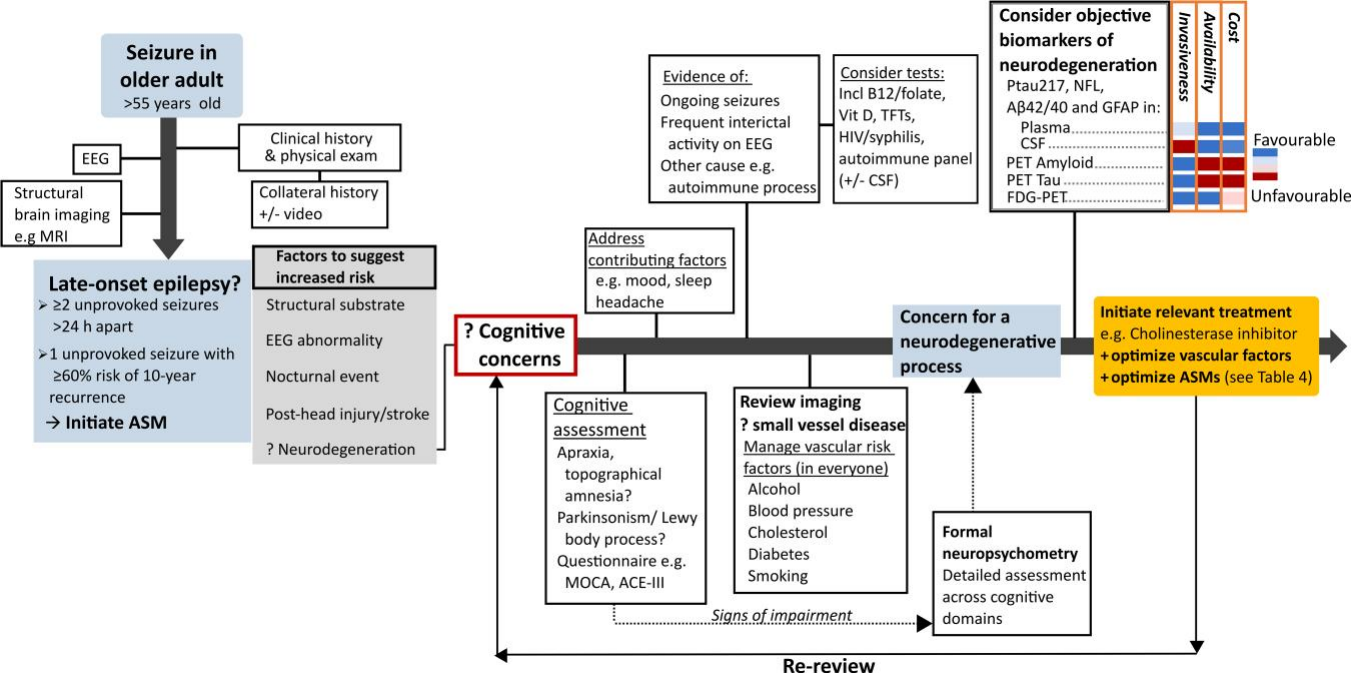
**Flow chart outlining a broad approach to late-onset epilepsy with potential underlying neurodegeneration**. A clinical approach to evaluating seizures and late-onset epilepsy in adults aged over 55 years. Clinical history, examination and initial EEG and MRI form the foundation for assessing seizures. Individuals diagnosed with epilepsy and potential underlying neurodegeneration should be closely reviewed with cognitive assessment, identifying vascular risk and other contributing factors. Concern for a neurodegenerative process may warrant specific investigations using fluid or imaging biomarkers of neurodegeneration. The biomarker choice should be informed by clinical suspicion for an underlying pathology and balanced by availability, invasiveness and cost of the investigation. Management, including anti-seizure medication (ASM) and risk factor optimization, should be reviewed regularly. ACE-III = Addenbrooke's Cognitive Examination, third edition; MOCA = Montreal Cognitive Assessment; TFT = thyrois function tests.

To help frame future discussions, we outline a broad approach to investigating later-onset epilepsy with potential underlying neurodegeneration (Fig. 2). Pragmatically, choosing which neurodegenerative biomarker to test will depend on clinical suspicion and consideration of availability, cost and invasiveness within the confines of individual healthcare settings. Balancing these factors, we suggest starting with P-tau217 for suspected AD and note NfL as a useful biomarker for detecting active neurodegeneration. Further investigations should then be based on initial results and other intersecting factors (Fig. 2).

### Electroencephalography as a biomarker

Tau pathology is associated with abnormal, detectable changes in the neurophysiological activity of the brain. Subclinical epileptiform activity (SEA) is observed in patients with AD with no history of overt epileptic seizures.^225^ While prevalence rates of scalp SEA in AD vary markedly (3%–54%),^226^ a substantial burden of mesial temporal epileptic activity was measured in two patients with AD using intracranial recordings with foramen ovale electrodes.^12^ Sleep has been identified as a prominent period for SEA, although more recent work has highlighted that the relationship between sleep, EEG and PET findings can be complex in people with AD.^227^

SEA may have clinical implications in AD. In a case-control study of 33 patients with AD and no history of seizures, the rate of detected SEA was four times higher compared with age-matched controls (42.4% versus 10.5%).^228^ Over a 3-year observational period, AD patients with SEA showed a 2.4-times faster cognitive decline on the Mini-Mental State Examination (MMSE) than AD patients without SEA (3.9 versus 1.6 points per year). The majority (90%) of epileptiform discharges in AD patients were detected during sleep.^228^ Similar findings were reported in another cohort of AD patients (*n* = 54) with no history of seizures.^229^ After 3 years of follow-up, cognitive decline was 1.5 times faster in AD patients with SEA compared with AD patients without SEA.

Electrophysiological changes associated with tau pathology are not necessarily specific for AD, being also found in FTD. Csernus and colleagues,^226^ for example, reported mesio-temporal SEA detected by deep brain electrodes in a patient with FTD with no seizure history and no EEG pathology detected on scalp recordings.

Another neurophysiological phenomenon associated with tau pathology are high frequency oscillations (HFOs) in the fast ripple frequency (FR-HFOs, 250–500 Hz), which were recently discovered in an animal model of AD.^230^ While HFOs can be seen under physiological conditions, pathological HFOs are associated across several neurological conditions. In epilepsy, HFOs have biological and clinical significance as a pathological electrical signal to prognosticate future seizure occurrence following brain injury in the rat LFPI model of TBI^231^; evaluate progression to status epilepticus in the rat 4-aminopyridine model^232^; identify the epileptogenic zone^233^ and predict epilepsy surgery outcome.^234^ HFOs may therefore be an electrophysiological biomarker of tau pathology. In the AD mouse models, HFOs are present and appear independent of Aβ deposits.^230^ This may be especially important in the context of cognition and memory consolidation as HFOs appear to be within the same frequency spectrum as other physiological markers of memory, for example hippocampal sharp wave-ripples.^235^

Further clinical and preclinical studies are needed to investigate whether or when HFOs can become a biomarker of tau pathology or associated seizures. The exact regulatory pathways involved in the interplay between brain injury, seizures, HFOs and tau pathologies also requires further elucidation.

## Starting treatment for epilepsy with potential underlying neurodegeneration using current anti-seizure medications

There is currently no evidence that a particular anti-seizure medication is more effective in individuals with epilepsy related to underlying neurodegeneration. Experiments using rodent models of AD have shown levetiracetam, topiramate and lamotrigine reduce aberrant epileptic activity, decrease tau phosphorylation, reduce Aβ plaque burden and improve cognition.^236-240^ Evidence is mixed, however, as high doses of levetiracetam did not improve cognitive decline in one investigation.^240^ Another study in a murine AD model showed no positive effect on epileptic activity with other anti-seizure medications including ethosuximide, gabapentin, phenytoin, pregabalin, valproic acid and vigabatrin.^237^ Valproic acid administered to AD rodent models did show benefit on epileptic discharges, Aβ plaque and tau phosphorylation levels,^241,242^ although there may be a specific effect for male mice.^243^ Evidence from the preclinical models has been reviewed extensively by Lehmann and colleagues.^244^

Two placebo-controlled randomized controlled cross-over trials using levetiracetam in individuals with mild cognitive impairment (MCI) or AD without a history of previous seizures have been completed.^245,246^ One of these studies suggested that levetiracetam may have a positive cognitive effect in individuals with EEG abnormalities at baseline.^245^ The second study could not evaluate primary end-point as recruitment was so adversely affected by the Coronavirus disease 2019 (COVID-19) pandemic. Both studies, though, showed a good safety profile for levetiracetam in people with dementia who had not experienced seizures.^218,219^ An earlier randomized, double-blind placebo controlled trial of valproic acid in mild-to-moderate AD over 24 months suggested worse cognitive outcomes in the treatment group as well as greater rate of volume loss on brain imaging.^247^

The decision, therefore, about which anti-seizure medication should first be tried in epilepsy associated with neurodegeneration is not straightforward, although the choice is governed by similar decision making to the initial choice of anti-seizure medication in older people.^248-251^ Principal options remain lamotrigine, levetiracetam and lacosamide.^248,252^ Intuitively, anti-seizure medications that may adversely affect cognition—for example topiramate, zonisamide, valproate (risk of hyperammonaemic encephalopathy)—should be avoided. Broad recommendations are provided in Table 4.

**Table 4:** Considerations and recommendation for initial anti-seizure medication in epilepsy with potential underlying neurodegeneration

Medication	Efficacy	Tolerability	Drug interactions	Key considerations in older individuals	Overall recommendation
Carbamazepine	Good	**Poor**	**High** (enzyme inducer)	High risk of adverse events; dizziness; drug–drug interactions	Generally avoided
Eslicarbazepine	Good	Moderate	Low–moderate	Similar to oxcarbazepine; risk of sodium imbalance, limited data in elderly	Limited data
Gabapentin	Moderate	Good	Minimal	Less effective; useful in frail/polymedicated patients	Possible alternative
Lacosamide	Good	Moderate	Low	Generally well tolerated but not extensively studied in older people	Reasonable alternative (limited data)
Lamotrigine^a^	Good	**Excellent**	Moderate	Considered as best-tolerated; effective; fewer cognitive effects; potentially helpful in comorbid low mood	Recommended
Levetiracetam^a^	Good	Good	**Minimal**	Effective; low interaction risk; behavioural effects in some	Recommended
Oxcarbazepine	Good	Moderate	Inducer of CYP enzymes	Risk of sodium imbalance; use with caution in older adults	Use with caution
Topiramate	Moderate	**Poor**	Low	Can cause cognitive dulling; weight loss; avoid in cognitively vulnerable patients	Often avoided
Valproic acid	Good	Poor	High	Tremor, sedation, weight gain, hepatotoxicity, risk of hyperammonaemic encephalopathy	Use only if essential

Anti-seizure medications (ASMs) are listed alphabetically with specific benefits and risks in bold. CYP = cytochrome P450.

^a^Consider as starting option.

## Discussion and future perspectives

The study of tau pathology in epilepsy provides insights into a potential mechanistic basis for network hyperexcitability in neurodegenerative diseases and, perhaps, epilepsy-related cognitive impairment. Early post-mortem studies of focal epilepsies identified greater than expected Braak scores.^24^ This was complemented by findings of increased tau pathology burden in temporal lobe surgical resection tissue, corresponding to pre- or postoperative cognitive measures.^21,139,149^ Transgenic murine models which overexpress tau produce longer epileptic discharges following kindling suggestive of an epileptogenic role of tau aggregation.^253^ Also, epilepsy-related conditions including Dravet syndrome,^135^ focal cortical dysplasia^23^ and post-traumatic epilepsy^127^ have been associated with abnormal tau aggregation.

Our current understanding of the interplay between tau pathology and epilepsy has deepened across biological and clinical dimensions, although there remains much that is poorly understood. It is important to formulate a path towards dissecting the role of the various conformation and PTMs of tau forms for the development of specific tau pathologies in the brain, such as neurofibrillary tangles, and the pathogenesis of neurocognitive, epilepsy disorders and neurodegeneration. Despite the increasing evidence that tau may have a pathological role in epilepsy, there is substantial heterogeneity across current studies, which include animal models, post-mortem analyses and clinical series. TLE has been most frequently examined, although other epilepsy syndromes are also implicated. Age may have an important moderating effect that needs further elucidating. Together, this highlights the need for future studies to apply standardized study designs and appropriate patient stratification to allow generalizability of results. An important direction must include the development of biomarkers that can better track tau forms *in vivo* dynamically, which would improve our understanding of the longitudinal processes involved in neurodegeneration, associated epilepsy and cognitive disorders across species.

Focusing on future clinical applications, measuring tau pathology *in vivo* offers a tangible biomarker for neuronal damage. Consideration must be given to the complexity of PTMs; tau hyperphosphorylation patterns and their interactions; dynamic temporospatial patterns and to establish whether specific PTMs are pathogenic or not. In comparing different studies, it is important to specify the exact phosphorylation sites being detected and quantified when referring to increases in phosphorylated or hyperphosphorylated tau. Without clear site-specific information, comparing findings across studies becomes challenging, as different methodologies and antibodies may target distinct phosphorylation sites, leading to inconsistencies in the literature. This lack of precision can also result in misinterpretation of findings, as tau phosphorylation is highly site dependent, with some modifications playing physiological roles while others drive pathological processes.

The recent development of fluid biomarkers of tau pathology, particularly blood-based measurements, represents a significant advance in this field.^254^ These tests are minimally invasive, affordable and scalable, which is ideal for routine clinical use enabling prediction, detection and monitoring of cognitive decline. Interpretation is challenging owing to the aforementioned complex patterns of post-translational tau modifications and the multiple factors that can regulate its expression and sequestration in the brain rather than blood. A multimodal biomarker approach including fluid, imaging, electrophysiological biomarkers, as well as clinical assessments and interventions will be essential to develop accurate predictive models for tracking and predicting neurocognitive or epilepsy outcomes. Validation of these tools and models using sufficiently phenotyped specimen repositories and multicentre large scale preclinical and clinical studies will also be necessary.

Together with objective, highly reliable cognitive testing that could be delivered remotely,^255^ tau-based multimodal biomarkers could be implemented into formal diagnostic criteria for epilepsy-related cognitive impairment, something that remains an ill-defined concept currently.^15^ Also, these assessments will be occurring within the milieu of a complex interplay of factors such as ageing, seizure semiology, risk from seizures—particularly head injury, anti-seizure medications and cardiovascular risk factors.^14,256^ All of these would need to be accounted for in any diagnostic criteria.

**Figure 3:**
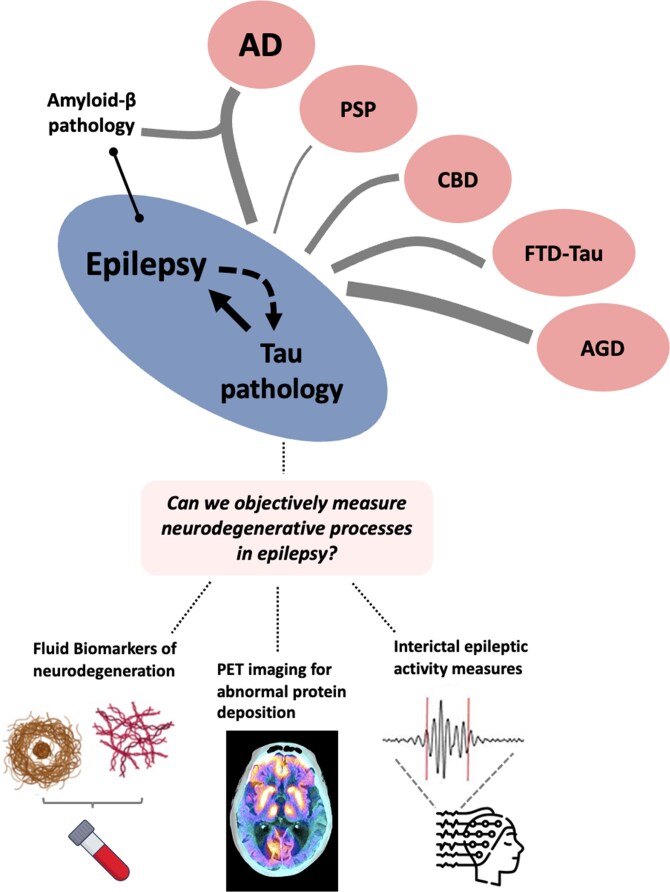
**Schematic figure depicting the bidirectional relationship between tau pathology and epilepsy.** Human post-mortem and surgical resection studies highlight the presence of tau pathology in individuals with epilepsy while experimental models demonstrate epileptogenic properties associated with greater tau burden. Such hyperexcitability is further exemplified by increased seizure prevalence observed in primary tauopathies (thickness of the line reflects seizure rates from a patient series of neuropathologically confirmed disease detailed in Table 2). An underlying neurodegenerative process in epilepsy may be detected *in vivo* by techniques such as fluid biomarkers, metabolic imaging and neurophysiology. AD = Alzheimer’s disease; AGD = argyrophilic grain disease; CBD = corticobasal degeneration; FTD = frontotemporal dementia; PSP = progressive supranuclear palsy.

In the relatively near future, longitudinal monitoring of cognition could be combined with biomarker studies to evaluate underlying pathological burden and provide a comprehensive clinical-biological picture. Integrating tau testing with other biomarkers, such as glial fibrillary acidic protein (GFAP) and NfL, may further enhance pathological accuracy. Blood biomarkers should also be integrated with EEG recording, particularly sleep EEG, to further stratify patient cohorts (schematic outlined in Fig. 3). Longitudinal, multimodal data with cognitive outcomes is imperative for our understanding into the causal relationship between tau pathology, seizures and neurodegeneration.

Beyond diagnostic testing, the potential treatment of epilepsy-related dementia with novel therapeutics targeting tau pathology is an exciting prospect. Immunotherapy against neuropathological proteinopathy has accelerated markedly in recent years for AD. The recent FDA approval of anti-amyloid immunotherapies, such as aducanumab and donanemab^92,257^ underscores the potential viability of such targeted therapies. Anti-tau agents have advanced from proof-of-concept studies to clinical trials (Supplementary Table 1) and could offer a similar treatment breakthrough in epilepsy-related cognitive decline.^258^ Sodium selenate, for example, reduces the accumulation of pathological phosphorylated tau in the brain by enhancing the activity of the protein phosphatase enzyme PP2A and has demonstrated anti-epileptogenic effects and disease-modifying effects in rat models of acquired epilepsy.^26,259^ A prospective double-blind randomized controlled trial of sodium selenate has commenced in adults with chronic drug-resistant TLE in Australia.^260^

Developing tau therapies has additional challenges compared with anti-amyloid treatment owing to complex biochemistry including six tau isoforms resulting in over 80 possible phosphorylation sites that might be targeted therapeutically. More recently, studies have focused on longer treatment periods (up to 5 years), recruiting participants at earlier disease stages and sample enrichment through screening for elevated tau levels. Outside of monoclonal antibodies, vaccines and antisense oligonucleotides are also entering Phase 1 and 2 trials.

To date, tau-targeting immunotherapies have not reached Phase 3 studies (Supplementary Table 1), primarily owing to failing to meet efficacy end-points in studies in AD/MCI and PSP. Inclusion criteria for older trials of monoclonal antibodies included MCI and mild, or even moderate AD (MMSE ≥ 15), and varying clinical diagnostic criteria. More recent studies have required more stringent inclusion criteria, including the Clinical Dementia Rating global score (CDR) 0.5–1, MMSE 20–30 and evidence of Aβ positivity. The primary outcome for all of these trials was change in the CDR-sum of boxes (over 1–2 years), with secondary outcomes covering a range of cognitive and functional measures as well as safety, pharmacokinetic and imaging measures. Despite the range of end-points, none of these studies met any of their primary or secondary efficacy end-points. Current ongoing trials of both monoclonal antibodies and antisense oligonucleotides use similarly restrictive inclusion criteria and have retained the CDR as their primary end-point. Trials in PSP have also failed to meet their primary and secondary end-points, resulting in increasingly restrictive inclusion criteria. The earliest monoclonal antibody trial (of BIIB092) included patients with both possible and probable PSP of all subtypes, without limitations on disease duration or severity (other than MMSE ≥ 20). Trials have since restricted recruitment to only PSP-Richardson’s syndrome (PSP-RS, classical PSP), criteria around symptom onset (<3/5 years), disability level (ability to walk 5/10 steps independently or PSP-rating scale < 40). The two trials that have completed did not meet either their primary (PSP-rating scale) nor secondary efficacy end-points. Ongoing studies of monoclonal antibodies (UCB0107) and antisense oligonucleotides (NIO572) have long (5-year) treatment periods with only safety/pharmacokinetic outcomes. Lastly, treatments targeting tau phosphorylation through upregulation of protein phosphatase 2 (sodium selenate) or inhibition of *O*-GlcNAcase (FNP-223), an enzyme which removes the sugar modification *O*-GlcNAc (*O*-linked *N*-acetylglucosamine) from intracellular proteins, have relatively restricted inclusion criteria (probable PSP-RS only for sodium selenate; PSP-RS, <3 years from symptom onset and the ability to walk 10 steps for FNP-223) to minimize the impact of variability in the study population on the study outcomes. The increasing homogeneity of populations studied may increase the likelihood of positive trials and reliability of data generated in the case of negative results. It may, however, also impact on the generalizability of the results and thus the translation from treatment of AD/PSP to epilepsy-related dementia.

Corollaries also apply. For example, anti-seizure medications are increasingly being explored as potential therapeutic options to help stabilize nerve cell networks in dementia.^245,246,261,262^ This would have advantages over the monoclonal antibody therapies including side effect profiles and cost. Notably, aducanumab has now been withdrawn, likely owing to the cost per quality-adjusted life year being nearly a million US dollars.^263^

In conclusion, understanding the role of tau pathology in epilepsy and related seizure conditions offers an exciting approach to better recognize, monitor and potentially manage cognitive decline and outcomes in people with epilepsy. There are important caveats including the complex process of establishing widely accepted diagnostic criteria as well as accounting for the biological intricacy of the tau protein when developing therapeutic agents. While challenges remain, integrating these approaches into clinical practice should ultimately improve the quality of life for people with epilepsy and offer broader therapeutic insights for people living with dementia.^264^

## Supplementary Material

awag108_Supplementary_Data
