# Supplementary material for: Tau pathology in epilepsy: emerging mechanisms and translational opportunities
Source: Brain. 2026 Mar 23;149(7):2250–72. doi: 10.1093/brain/awag108 (PMC13337244; doi:10.1093/brain/awag108)
Supplement: awag108_Supplementary_Data [file awag108_supplementary_data.pdf]

Supplementary Table 1. Clinical trials using therapies focused on reducing tau pathology

Agent	Synonyms	Tau epitope	Subject and study phase	Company	ClinicalTrials.gov ID	Trial status
AADvac-1	Axon peptide 108 conjugated to KLH	294-305	Mild to moderate Alzheimer's Disease (Phase 2), Progressive Nonfluent Aphasia (Phase 1)	Axon Neuroscience SE	NCT02579252	Completed 2019. Further Phase 2B planned. No results announced
			Progressive Supranuclear Palsy (Phase 2 platform)			Part of curePSP's platform trial. No details currently available
ACI-35	JNJ-2056	p-Ser396,404	Early Alzheimer's Disease (Phase 2)	AC Immune SA, Janssen	NCT04445831	Completed. No efficacy data currently available.
					ISRCTN78730935	Recruiting, estimated completion 2031
UCB0107	Bepranemab, Antibody D	235-246	mild AD/MCI (Phase 2)	UCB Biopharma, Genentech	NCT04867616	Recruitment complete
			Progressive Supranuclear Palsy (Phase 1 and OLE)		NCT04185415, NCT04658199	Phase 1 complete, no results. OLE estimated completion 2027.

BIIB092	Gosuranemab, BMS986168, IPN007	8-19	Mild Alzheimer's Disease (Phase 2)<br><br>Progressive Supranuclear Palsy (Phase 2)	Biogen, Bristol-Meyers Squibb	NCT03352557<br><br>NCT03068468	Discontinued: no efficacy (2021)<br><br>Discontinued: no efficacy (2019)
BIIB080(CELIA)	IONIS-MAPTRx, ISIS 814907	Antisense Oligonucleotide targeting *MAPT* mRNA	Mild Cognitive Impairment (Phase 2)	Biogen, IONIS Pharmaceuticals	NCT05399888	Estimated completion Q4 2026
CN2-8E12	Tilavonemab, ABBV-8E12, HJ9.3	25-30	Early Alzheimer's Disease (Phase 2)<br><br>Progressive Supranuclear Palsy (Phase 2)	AbbVie, C2N Diagnostics, LLC	NCT03712787<br><br>NCT02985879	Completed no efficacy (2021)<br><br>Discontinued, no efficacy (2019)
E2814		HVPGG motif in the microtubule binding domain, (299-303, 362-366)	Healthy or MCI participants with Alzheimer's disease genetic mutation (DIAN-TU Phase 2)	Eisai Co., Ltd.	NCT05269394	Estimated completion Q2 2028
LY3303560	Zagotenemab	conformational	Early Alzheimer's Disease (Phase 2)	Eli Lilly & Co.	NCT03518073	Completed: No efficacy (2021)

		(7-9, 312-342)			NCT05063539	Completed(2024) Primary endpoint not met. Several secondary endpoints showed greater decline in high dose group compared to placebo.
JNJ-63733657	Posdinemab	mid-region, pT217	Early Alzheimer's Disease (Phase 2)	Janssen	NCT04619420	Estimated completion Q1 2026
RO7105705	Semorinemab, MTAU9937A, RG6100	N-terminus of all six isoforms	Moderate Alzheimer's Disease (Phase 2)	AC Immune SA, Genentech	NCT03289143	Completed. No efficacy (2020)
Lu AF87908		p-Ser396	Healthy, Alzheimer's Disease (Phase 1)	H. Lundbeck A/S	NCT04149860	Completed Q3 2023 (results unknown)
PNT001		cis-pT231	Healthy participants (Phase 1), Acute traumatic brain injury (Phase 1)	Pinteon Therapeutics	NCT04096287	Terminated 2021 (non-safety reasons)
NIO752		Antisense Oligonucleotide targeting *MAPT*	Alzheimer's disease (Phase 1)	Novartis Pharmaceuticals	NCT05469360	Recruiting, estimated completion 2027
			Progressive Supranuclear Palsy			Completed Q4 2024, results not

		mRNA	(Phase 1)		NCT04539041	yet released
FNP-223		Inhibition of O-GlcNAcase to reduce tau phosphorylation	Progressive Supranuclear Palsy (Phase 2)	Ferrer Internacional S. A.	NCT06355531	Recruiting, estimated completion Q4 2026.
Sodium Selenate	Selenate, VEL015	Enhance PP2A activity to reduce pathological brain h-tau	Alzheimer's Disease (Phase 2)	Monash University	ACTRN12611001200976	Completed: No efficacy (2018)
			bvFTD (Phase 1 – completed, Phase 2 ongoing)		ACTRN12620000236998, ACTRN12617001218381	Completed 2021, estimated completion Q2 2027
			PSP (Phase 2 ongoing)		ACTRN12620001254987	Estimated completion Q2 2027
			MTLE (Phase 2 – ongoing)		ACTRN12623000446662	Estimated completion Q1 2029
